# Clinical Evaluation of Duchenne Muscular Dystrophy Severity Using Ultrasound Small-Window Entropy Imaging

**DOI:** 10.3390/e22070715

**Published:** 2020-06-28

**Authors:** Dong Yan, Qiang Li, Chia-Wei Lin, Jeng-Yi Shieh, Wen-Chin Weng, Po-Hsiang Tsui

**Affiliations:** 1 School of Microelectronics, Tianjin University, Tianjin 300072, China; yd_beiyang@tju.edu.cn (D.Y.); liqiang@tju.edu.cn (Q.L.); 2 Department of Physical Medicine and Rehabilitation, National Taiwan University Hospital Hsin-Chu Branch, Hsin-Chu 30059, Taiwan; chiaweionly@gmail.com; 3 Department of Physical Medicine and Rehabilitation, National Taiwan University Hospital, Taipei 100229, Taiwan; jyshieh@ntu.edu.tw; 4 Department of Pediatrics, National Taiwan University Hospital, and College of Medicine, National Taiwan University, Taipei 100233, Taiwan; 5 Department of Pediatric Neurology, National Taiwan University Children’s Hospital, Taipei 100226, Taiwan; 6 Department of Medical Imaging and Radiological Sciences, College of Medicine, Chang Gung University, Taoyuan 33302, Taiwan; 7 Medical Imaging Research Center, Institute for Radiological Research, Chang Gung University and Chang Gung Memorial Hospital at Linkou, Taoyuan 33302, Taiwan; 8 Department of Medical Imaging and Intervention, Chang Gung Memorial Hospital at Linkou, Taoyuan 33305, Taiwan

**Keywords:** Duchenne muscular dystrophy, entropy, ultrasound, backscattered signals

## Abstract

Information entropy of ultrasound imaging recently receives much attention in the diagnosis of Duchenne muscular dystrophy (DMD). DMD is the most common muscular disorder; patients lose their ambulation in the later stages of the disease. Ultrasound imaging enables routine examinations and the follow-up of patients with DMD. Conventionally, the probability distribution of the received backscattered echo signals can be described using statistical models for ultrasound parametric imaging to characterize muscle tissue. Small-window entropy imaging is an efficient nonmodel-based approach to analyzing the backscattered statistical properties. This study explored the feasibility of using ultrasound small-window entropy imaging in evaluating the severity of DMD. A total of 85 participants were recruited. For each patient, ultrasound scans of the gastrocnemius were performed to acquire raw image data for B-mode and small-window entropy imaging, which were compared with clinical diagnoses of DMD by using the receiver operating characteristic curve. The results indicated that entropy imaging can visualize changes in the information uncertainty of ultrasound backscattered signals. The median with interquartile range (IQR) of the entropy value was 4.99 (IQR: 4.98–5.00) for the control group, 5.04 (IQR: 5.01–5.05) for stage 1 patients, 5.07 (IQR: 5.06–5.07) for stage 2 patients, and 5.07 (IQR: 5.06–5.07) for stage 3 patients. The diagnostic accuracies were 89.41%, 87.06%, and 72.94% for ≥stage 1, ≥stage 2, and ≥stage 3, respectively. Comparisons with previous studies revealed that the small-window entropy imaging technique exhibits higher diagnostic performance than conventional methods. Its further development is recommended for potential use in clinical evaluations and the follow-up of patients with DMD.

## 1. Introduction

Information entropy of ultrasound imaging recently receives much attention in the diagnosis of Duchenne muscular dystrophy (DMD). DMD is the most common muscular disorder caused by mutations in the dystrophin gene [1] and results in absent or insufficient functional dystrophin, which leads to reduced sarcolemma stability and rendering the muscle fibers vulnerable to mechanical stretching-induced injury [2]. As a consequence, repeated contraction leads to necrosis and the regeneration of muscle fibers, which are gradually replaced by fat and fibrous tissue. This disease is primarily an X-linked condition affecting males; however, some female carriers exhibit symptoms of the disorder, but usually with a milder phenotype [3]. Because of regional and ethnic differences, the estimated incidence is approximately 1 in 5000–10,000 live male births [4,5,6]. Boys with DMD may exhibit symptoms such as abnormal gait, weakened proximal muscles, and calf muscle pseudohypertrophy at age 3–5 years [7,8]. Patients with DMD inevitably develop a loss of mobility, respiratory and cardiac deterioration as a consequence of the dystrophic changes of muscle, and typically die from respiratory and cardiac complications by the age of 30 [9].

Currently, no curative therapy is available for treating DMD; therefore, early detection and effective health care, rehabilitation, and psychosocial management are essential [10,11,12]. However, considerable progress has been made recently in terms of genetic approaches [13]; some drugs have also been conditionally approved for the treatment of patients with DMD [14]. This implies that reliable and noninvasive approaches to evaluating the progression of DMD and treatment efficacy are required. The North Star Ambulatory Assessment [15] and timed function tests, including the 6-minute walk test, time to climb 4 stairs, time to stand or 10-meter walk [16] are typical assessments of function during the ambulatory period. Although these outcome measures are clinically meaningful and valuable, their sensitivity is often limited by the effort and mood of children with DMD without objective assessment of muscle pathologic change [17].

Ultrasound and magnetic resonance imaging (MRI) are two of the commonest and widely used noninvasive methods for muscle tissue examination [18,19]. Compared with MRI, ultrasound imaging enables friendlier and safer routine scans and follow-up for pediatric patients [20]. Studies have revealed that quantitative muscle ultrasound can detect DMD progression [21,22]. Fat infiltration and fibrosis formation increase the intensity of the backscattered echo [23], indicating that ultrasound backscattering may provide useful information associated with changes in muscle microstructures for DMD diagnosis. Considering the random nature of ultrasound backscattering, the probability distribution of the backscattered envelope (the echo amplitude) has been explored and demonstrated to be useful in characterizing tissues [24]. Previously, the Nakagami statistical distribution was applied to modeling the backscattered statistics as an evaluation method of ambulatory function in patients with DMD [25]. However, the prerequisite for using the statistical distribution is that the echo data must follow the model used [26]; it is difficult to satisfy the aforementioned condition in practice, because the properties of backscattered signals depend on system characteristics, software settings, and signal/image processing. This limitation has encouraged researchers to pursue a more flexible solution for describing backscattering information, without considering the distribution nature of the echo data.

Among all possibilities, Shannon entropy (a measure of information uncertainty [27]) fulfills the aforementioned requirement. Hughes first introduced the concept of entropy in the field of ultrasound imaging, indicating that entropy can be used to quantitatively characterize changes in the microstructures of scattering media [28,29,30]. Furthermore, entropy has been reported to be a non-model-based statistical parameter that is proportional to the Nakagami parameter and correlates with backscattered statistics [31]. In particular, information entropy has been applied to ultrasound parametric imaging, allowing the use of the small-window technique to visualize the statistical properties of backscattered signals for tissue characterization with improved image resolution [32,33]. For these reasons, we explored the feasibility of using ultrasound small-window entropy imaging in evaluating the severity of the dystrophic process in patients with DMD.

## 2. Materials and Methods

### 2.1. Study Population

The Institutional Review Board of National Taiwan University Hospital (NTUH) approved the study and allowed the reuse of the database collected in a previous study [34]. A total of 85 participants (*n* = 85) aged between 2 and 24 years provided written informed consent, and the experimental methods were conducted according to the approved guidelines. All DMD patients (*n* = 73) were recruited from the joint clinics of neuromuscular disorders in the Department of Pediatrics, NTUH. The clinical manifestations of each patient were consistent with DMD, and diagnoses had been confirmed according to muscle biopsies (revealing absent dystrophin) and/or genetic testing. On the basis of a review report [10], the severity of DMD was classified into three stages: stage 1 (presymptomatic, early ambulatory, and late ambulatory), stage 2 (early non-ambulatory), and stage 3 (late non-ambulatory). Seventy-three patients (*n* = 73) were recruited (stage 1: *n* = 41; stage 2: *n* = 20; stage 3: *n* = 12). Twelve children (*n* = 12) with no history of weakness or neuromuscular disorders were also recruited as controls. The demographic data of participants and stage definitions are summarized in our previous study [34].

### 2.2. Ultrasound Examination

A commercial clinical ultrasound system (Model 3000; Terason, Burlington, MA, USA) equipped with a linear array transducer (Model 12L5A; Terason) was used for ultrasound scans on the patients with DMD. The central frequency, pulse length, and beam width of the transducer were 7 MHz, 0.7 mm, and 1.2 mm, respectively. All participants underwent a standard-care ultrasound examination of the gastrocnemius using the sagittal scanning approach. For each participant, three valid scans (i.e., no acoustic shadowing artifacts and the exclusion of large vessels in the region of analysis) were performed by a skilled physician. The focus and depth were set at 2 and 4 cm, respectively. The gain index of the Terason system was set at 6, corresponding to a signal-to-noise ratio of approximately 30 dB, which was obtained from the calibrations performed in the previous study [35]. Raw image data obtained from each valid scan, consisting of 128 backscattered radiofrequency (RF) signals at a sampling rate of 30 MHz, were used for offline data processing in MATLAB, including ultrasound B-mode and small-window entropy imaging.

### 2.3. Entropy Imaging Algorithm

**Figure 1:**
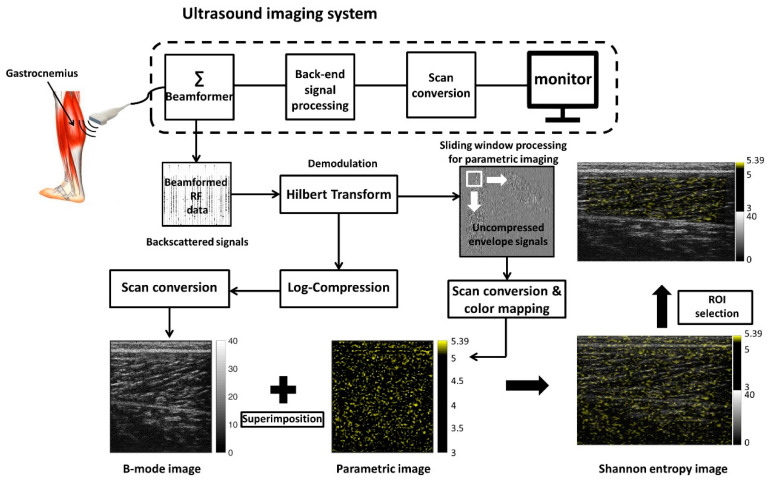
Algorithms for ultrasound B-mode and entropy imaging. The uncompressed envelope data were processed using the sliding window technique. The side length of the window was set as one time the pulse length, to acquire local data points for estimating the entropy values. RF: radiofrequency.

The algorithms for ultrasound B-mode and entropy imaging [32] are illustrated in Figure 1. For the data of each raw image, the absolute values of the Hilbert transform of backscattered RF signals were calculated to obtain the envelope image. Using the logarithm-compressed envelope, which provides different grayscales according to its value at a dynamic range of 40 dB, the B-mode image was formed. The uncompressed envelope data were used for small-window entropy imaging according to the following steps: (a) a small-square window was set up in the upper-left corner of the data with a side length of one time the pulse length of the transducer (0.7 mm) to collect uncompressed envelope data; (b) the envelope data were normalized, and the probability distribution of the envelope data within the window was constructed using a statistical histogram (bins = 50) for estimating the Shannon entropy, using the following equation:

(1) $$H_{C}=-\overset{n}{\underset{i=1}{\sum }}w\left(y_{i}\right)\log_{2}\left[w\left(y_{i}\right)\right]$$

where *y*_i_ is the discrete random variable of the envelope data, *w*(*y*_i_) represents the probability value, and *n* indicates the number of bins; then, the estimated entropy value was assigned as a new pixel corresponding to the window location; (c) subsequently, the window, with a window overlap ratio of 50%, to provide a tradeoff between the parametric image resolution and computational time [32], was slid throughout the entire envelope image to calculate local entropy values (according to the step (b)) for generating a parametric map; (d) a two-dimensional linear interpolation was performed to obtain an entropy parametric map, with the same size as the uncompressed envelope data [36], which was displayed in a pseudocolor and superimposed onto the B-mode image, to reveal both the anatomical and backscattering information. Finally, the region of interest (ROI) corresponding to the gastrocnemius was manually chosen on the image to calculate the average entropy value.

The ROI selection was handled by a pediatric neurologist. To reduce the bias in averaging the entropy values in the ROI, choosing an ROI that satisfies the coverage of the whole gastrocnemius was used as a basic rule in this study. For each participant, the final entropy value was obtained by the average of three valid scans.

### 2.4. Statistical Analysis

The envelope amplitude and entropy values, as a function of DMD stage, are expressed as vertical box and dotted plots, which exhibit the median, interquartile range (IQR; being equal to the difference between the third quartile and the first quartile), data distribution, and outliers. The Spearman rank correlation coefficient *r* and the probability value *p* were calculated for evaluating the correlation between the parameter values (envelope amplitude and entropy) and DMD stage. The receiver operating characteristic (ROC) curve with a 95% confidence interval (CI) was used to evaluate the performances for diagnosing different DMD stages. The ROC curve was created by plotting the true positive rate against the false positive rate at various threshold settings. The optimal cutoff value for diagnosing each DMD stage was determined by the point maximizing the Youden function, which is the difference between true positive rate and false positive rate over all possible cutoff values [37]. The area under the ROC curve (AUROC), sensitivity, specificity, accuracy, and other statistical results were then reported. A *p*-value of <0.05 was considered statistically significant. All statistical analyses were performed using SigmaPlot Version 12.0 (Systat Software, Inc., CA, USA). 

## 3. Results

**Figure 2:**
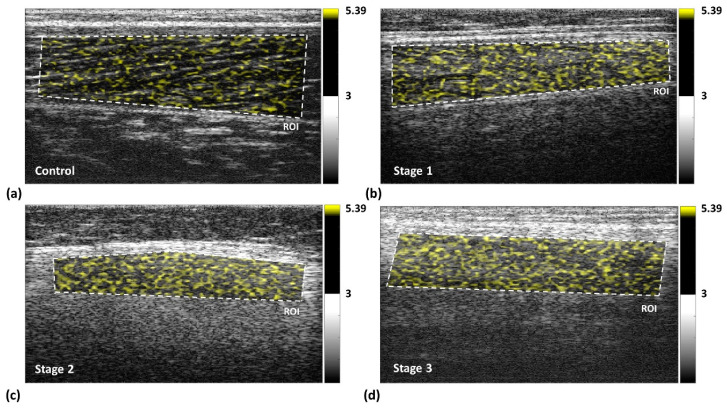
Typical images measured at different Duchenne muscular dystrophy (DMD) stages. (**a**) normal control; (**b**) stage 1; (**c**) stage 2; (**d**) stage 3. The dotted lines indicate the regions of interest (ROIs) corresponding to the gastrocnemius. Image brightness increased between the control group and the stage 3 group, representing a corresponding entropy value increase.

**Figure 3:**
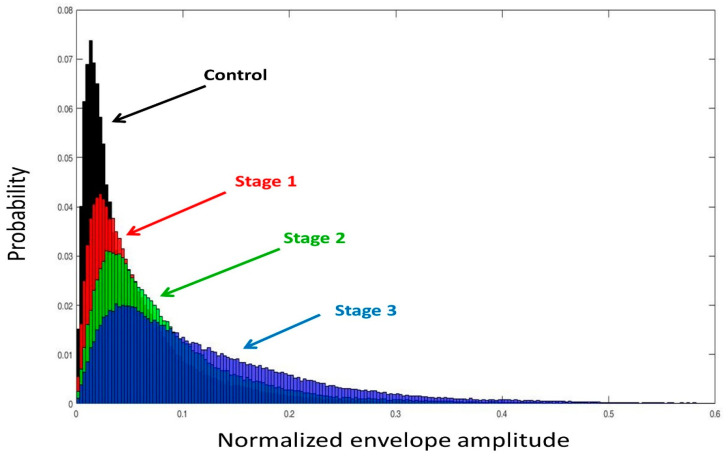
Probability distributions of ultrasound backscattered signals measured in the ROIs for different DMD stages. The probability distribution was described using a statistical histogram (bins = 50); it varied with the severity of DMD.

Typical images representing different DMD stages are depicted in Figure 2. The dotted lines indicate the ROIs corresponding to the gastrocnemius for ultrasound entropy imaging. Observations on the entropy values obtained from three valid scans for each individual subject showed that the proposed rule for ROI selection ensured the maximum difference of entropy between three valid scans ≤0.02. The image brightness increased from the control group to stage 3, indicating that the entropy value and the probability distribution of ultrasound backscattered signals vary with the severity of DMD (Figure 3).

**Figure 4:**
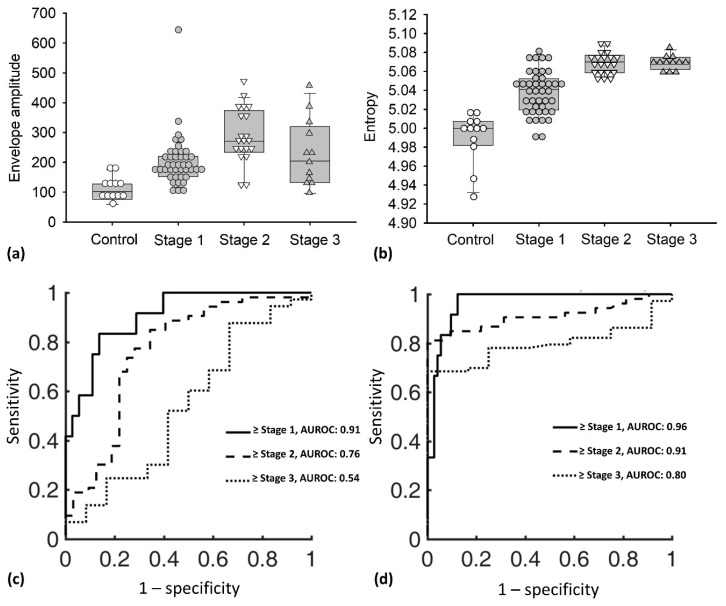
(**a**,**b**) Envelope amplitude and entropy values as a function of DMD stage. Data were expressed by vertical box and dotted plots, which revealed the median, interquartile range (IQR), data distribution, and outliers. The entropy value increased as the DMD stage advanced (*r* = 0.76; *p* < 0.05), and the envelope amplitude also showed a similar trend (*r* = 0.49; *p* < 0.05). (**c**) and (**d**) AUROCs for diagnosing different DMD stages using the B-mode and entropy images. Compared with the B-scan, ultrasound entropy imaging could detect early stage DMD with improved diagnostic performance; it also performed well in detecting the difference between ambulatory and non-ambulatory stages.

Figure 4a,b exhibits the box plots with dot density, which reveal the positions of each envelope amplitude and entropy data point. Evidently, the envelope amplitude increased as the DMD stage advanced (*r* = 0.49; *p* < 0.05); the median (IQR) was 102.32 (IQR: 75.90–125.44) for the control, 178.99 (IQR: 158.22–218.73) for stage 1, 271.08 (IQR: 236.65–363.11) for stage 2, and 204.97 (IQR: 135.08–300.83) for stage 3. Ultrasound entropy also increased as DMD stages progressed (*r* = 0.76; *p* < 0.05); the median (IQR) was 4.99 (IQR: 4.98–5.00) for the control, 5.04 (IQR: 5.01–5.05) for stage 1, 5.07 (IQR: 5.06–5.07) for stage 2, and 5.07 (IQR: 5.06–5.07) for stage 3. The AUROCs (95% CI) for diagnosing different DMD stages are shown in Figure 4c,d. The AUROCs obtained from using the B-scan to calculate the envelope amplitude were 0.91 (0.79–1), 0.76 (0.66–0.86), and 0.54 (0.36–0.72) for ≥stage 1, ≥stage 2, and ≥stage 3, respectively (the diagnostic accuracies were 85.88% for ≥stage 1, 75.29% for ≥stage 2, and 52.94% for ≥stage 3), and those of entropy were 0.96 (0.89–1), 0.91 (0.85–0.97), and 0.80 (0.68–0.91) for ≥stage 1, ≥stage 2, and ≥stage 3, respectively (the diagnostic accuracies were 89.41% for ≥stage 1, 87.06% for ≥stage 2, and 72.94% for ≥stage 3). Table 1 and Table 2 show the other statistical results obtained from the ROC analysis, including cutoff value, sensitivity, specificity, positive likelihood ratio, negative likelihood ratio, positive predictive value, and negative predictive value, representing that ultrasound entropy imaging outperformed conventional B-scan in evaluating the severity of DMD.

## 4. Discussion

In DMD, the progression of two critical periods should be noted: the first refers to the period before amyotrophia occurs and the second is when patients lose their ambulation. However, free-acting capability is apparently a critical index representing the effect of DMD on patients and their families. For this reason, the major aim of DMD treatment is the prolongation of walking function [38]. In most cases, neuromuscular specialists assess and characterize each patient’s unique disease trajectory using validated assessment tools and their clinical experience, aiming to establish a patient’s expected clinical course [10]. Because of advances in medical technologies, ultrasound imaging has become the preferred method for clinicians to follow up DMD patients, because it serves as a real-time point-of-care tool. Ultrasound imaging can also provide further quantitative information associated with the echo intensity to aid DMD evaluations [39,40]. To satisfy the requirement for evaluating the walking function of patients with DMD, an emerging research trend involves using statistical distributions to model the backscattered statistics for characterizing tissue microstructures, which are highly correlated with the behavior of ultrasound backscattering [24].

As reviewed in the Introduction, ultrasound Nakagami parametric imaging has been applied to imaging the backscattered statistics measured from the gastrocnemius [25]. Variation in the Nakagami parameter from 0 to 1 indicates a change in the envelope statistics from a pre-Rayleigh to a Rayleigh distribution; a Nakagami parameter higher than 1 indicates that the backscattered statistics represent post-Rayleigh distributions [25]. The Nakagami parameter increased and remained close to 1 when the DMD progressed to stage 4; the performance of diagnosing the walking function of the patients with DMD was also acceptable (AUROC: 0.89; accuracy: 85.52%; sensitivity: 76.31%; specificity: 94.73%) [25]. In this study, we used information entropy, a simpler and more effective parameter, as the non-model-based solution to analyze the uncertainty and complexity of ultrasound backscattered signals. We also followed the algorithm of ultrasound parametric imaging to construct the entropy map to image the backscattering information of DMD. According to our findings, the entropy value was a monotonically increasing function of the DMD stage, and gradually entered a plateau phase when DMD was at stages 2 and 3, representing that distinguishing stages 2 and 3 is difficult. However, clinical treatments and managements of DMD actually need early detection and evaluations of the walking function (at stages 1 and 2), and ultrasound entropy may be a qualified imaging biomarker to satisfy the above purposes. Evidently, ultrasound entropy imaging was able to detect early stage DMD with improved diagnostic performance (AUROC: 0.96; accuracy: 89.41%; sensitivity: 100%; specificity: 87.67%). Moreover, ultrasound entropy values accurately detected the difference between ambulatory and non-ambulatory stages (AUROC: 0.91; accuracy: 87.06%; sensitivity: 84.91%; specificity: 90.63%). Several studies have revealed that fatty and connective tissues in the muscles of patients with DMD cause strong echoes [21,22,40]. This can be regarded as the behavior of constructive wave interference to induce changes in the backscattered statistics from the pre-Rayleigh to the Rayleigh distribution, corresponding to the increase in the signal uncertainty [31,33]. This can explain why the Nakagami and entropy values increase with the severity of DMD. More importantly, ultrasound entropy imaging exhibited improved diagnostic performance for DMD evaluations compared with the Nakagami parameter proposed previously.

The improved diagnostic performance of ultrasound entropy imaging may be attributed to the suppression of boundary artifacts during sliding window processing. Entropy and Nakagami images differ in that entropy allows the use of a small sliding window for parametric imaging. This advantage enables an effective reduction in the appearance of boundary artifacts in ultrasound parametric imaging constructed using the sliding window processing technique, as explained in detail previously [32]. Briefly, as the sliding window moves across the interface, the window acquires not only the backscattered signals returned from the interface, but also those from the tissue parenchyma. The difference in the echo amplitude of the interface and tissue parenchyma tends to lead to underestimation of the parameter, generating a boundary artifact. The simplest approach to suppressing boundary artifacts is to use a small window for parametric imaging. However, the distribution parameters typically require a relatively large window for parametric imaging, because a sufficient sample size is necessary for stable parameter estimation. Unlike the distribution parameters, information entropy is a relative measure of signal uncertainty, not a model-based parameter or an absolute physical estimate. Therefore, entropy calculated using fewer data points acquired from a small window for scatterer characterization is allowed and feasible [32]. On the other hand, the offline processing time for ultrasound entropy imaging of one image raw data was < 1 sec (operating environment: Windows 10; RAM: 8 GB; CPU: Intel^®^ Core^TM^ i3-8100 at 3.6 GHz), implying the possibility of real-time capability if the algorithm is combined with an ultrasound system.

The limited dynamic range of information entropy is the major limitation in practice, although this does not affect the statistical significance of the results. The dynamic range of information entropy must be enlarged to facilitate the color mapping and visualization of parametric imaging as well as to improve its sensitivity. Probably, using phantoms with different scatterer properties to find a theoretically dynamic range of entropy as the calibration reference for parameter normalization may be a feasible method. Moreover, using ultrasound entropy imaging to follow up on patients with DMD is an area for future research, which could provide possibilities to predict the progression of walking function in these patients. Prior to using entropy imaging as a reliable follow-up tool of DMD, a large-scale clinical validation is still necessary. Using different datasets for training and tests are also needed to investigate the power of prediction through ultrasound entropy imaging.

## 5. Conclusions

In this study, we investigated the performance of ultrasound small-window entropy imaging in evaluating the clinical severity of symptoms in patients with DMD. The results revealed that entropy imaging can visualize changes in the information uncertainty of ultrasound backscattered signals during the progression of DMD. In particular, entropy value performed well in detecting the early stages of DMD; entropy values were also highly correlated with walking function in patients with DMD. Compared with conventional B-scan and model-based methods, entropy imaging, constructed using the small-window technique, exhibits great potential, and we recommended its further development for the clinical evaluations and follow-up of patients with DMD. 

## Figures and Tables

**Table 1:** Clinical performance of ultrasound B-mode imaging (envelope amplitude) in evaluating the severity of DMD.

Clinical Severity	≥Stage 1	≥Stage 2	≥Stage 3
**Cutoff value**	128.22	212.86	183.04
**Sensitivity**	83.33	77.36	52.05
**Specificity**	86.3	71.88	58.33
**Accuracy**	85.88	75.29	52.94
**LR+**	6.08	2.75	1.25
**LR-**	0	0.32	0.82
**PPV, %**	50	82	88.37
**NPV, %**	96.92	65.71	16.67
**AUROC (95% CI)**	0.91 (0.79–1)	0.76 (0.66–0.86)	0.54 (0.36–0.72)

LR+: positive likelihood ratio, LR−: negative likelihood ratio, PPV: positive predictive value, NPV: negative predictive value, AUROC: area under the receiver operating characteristics curve.

**Table 2:** Clinical performance of ultrasound entropy imaging in evaluating the severity of DMD.

Clinical Severity	≥Stage 1	≥Stage 2	≥Stage 3
**Cutoff value**	5.01	5.05	5.05
**Sensitivity**	100	84.91	68.49
**Specificity**	87.67	90.63	100
**Accuracy**	89.41	87.06	72.94
**LR+**	8.11	9.06	6.55
**LR-**	0	0.17	0.32
**PPV, %**	57.14	93.75	100
**NPV, %**	100	78.38	34.29
**AUROC (95% CI)**	0.96 (0.89–1)	0.91 (0.85–0.97)	0.80 (0.68–0.91)
